# The crux of menopausal hormone therapy: dose, route, and age

**DOI:** 10.1016/j.rpth.2023.102269

**Published:** 2023-11-23

**Authors:** Linzi M. Hobbs, Lisa M. Baumann Kreuziger

**Affiliations:** 1 Department of Medicine, Medical College of Wisconsin, Milwaukee, Wisconsin, USA; 2 Versiti Blood Research Institute, Milwaukee, Wisconsin, USA

Women were first prescribed menopausal hormone therapy (MHT) in the 1960s as high-dose oral estrogen [1]. An increased risk of endometrial cancer was subsequently identified in people taking estrogen but not in people prescribed combined estrogen and progesterone. Use of estrogen-progesterone formulations soared in the 1990s because epidemiologic studies suggested that MHT reduced hot flashes and prevented coronary heart disease, osteoporosis, and dementia [1]. Two randomized, double-blind, placebo-controlled trials were subsequently launched to evaluate the potential benefit of MHT on cardiovascular disease and risk of treatment. The Heart and Estrogen-Progestin Replacement Study found no overall impact on cardiovascular events and a 3-fold increase in venous thromboembolism (VTE) among patients with coronary artery disease using MHT [2] (Figure). The authors recommended that people should not start MHT for secondary prevention of cardiovascular disease, but people already receiving it should continue treatment due to decreased cardiovascular events after years of therapy. The Women’s Health Initiative (WHI), however, showed that MHT was associated with greater risks than benefits as it doubled the risk of VTE [4,5]. Within a year of the WHI results being released, MHT prescriptions plummeted by 43% to 46% [6,7]. Although studies have suggested the safety of alternative MHT preparations, providers remain reluctant to prescribe MHT to ease menopausal symptoms.

**Figure:**
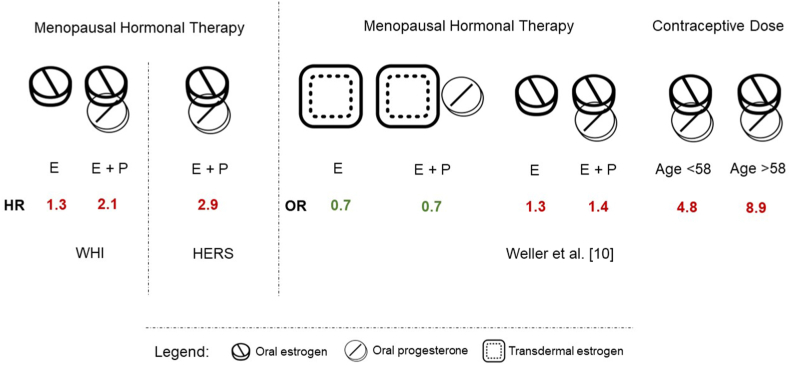
The risk of venous thromboembolism (VTE) associated with different hormone doses, routes of administration, and patient age. The Women’s Health Initiative (WHI), Heart and Estrogen-Progestin Replacement Study (HERS), and Weller et al. [3] showed increased risk of VTE associated with estrogen (E) or combined estrogen and progesterone (E + P) use. Highest VTE risk occurred with combined oral contraceptives in women aged >58 years. Transdermal estrogen was not associated with increased VTE risk. Red text denotes statistically significant higher risk, and green text denotes no increased risk. HR, hazard ratio; OR, odds ratio.

Recent studies from Europe have found that oral estrogens, but not transdermal estrogens, were associated with VTE risk [[8], [9], [10]]. A recent *Research and Practice in Thrombosis and Haemostasis* article by Weller et al. [3] is the first nested case-control study in the United States to investigate the risk of VTE associated with hormonal therapy in women aged 50 to 64 years. They compared formulations used for MHT to combined oral contraceptives, the route of administration (transdermal vs oral), and the age of use. Similar to the European studies, Weller et al. [3] found that transdermal estrogen was not associated with an increased risk of VTE (Figure). Interestingly, oral contraceptive use was associated with a 4-fold higher risk of VTE compared to that in the oral MHT group and a 5-fold higher risk compared to that among people not taking hormones. When comparing types of estrogen, estradiol had the lowest risk and ethinyl estradiol had the highest risk.

There are some limitations to this study inherent to administrative databases. Sensitivity of International Classification of Diseases, 10th Revision, codes for diagnosis of VTE events while hospitalized was 100%, while specificity was 79.3% [11]. One way to improve accuracy, as done by Weller et al. [3], is to combine International Classification of Diseases, 10th Revision, codes with filling of an anticoagulant prescription. Another issue is a potential bias in when hormone therapy was prescribed. Given the results of the WHI, people believed to be at higher risk of VTE would be less likely to be prescribed MHT. Inclusion of higher-risk patients in the control group would have decreased the VTE risk between the exposure groups (ie, biased the results to the null). This bias would likely have not affected the overall results given the large cohort but must be considered with the smaller subgroups.

When comparing the study by Weller et al. [3] to the WHI, one striking difference is the age of participants. Majority of women in the WHI were aged ≥60 years, with only a third of participants in their 50s. Weller et al. [3] included women aged 50 to 64 years as the average age of menopause is 51 years. Therefore, the risk estimates established in the current study are more applicable to current prescribing practices. The study importantly showed that people aged >58 years taking contraceptive doses experienced the highest VTE risk (Figure). Therefore, the risk of VTE is associated not only with hormone dose but also with the person’s age.

Overall, this study is the first in the United States to investigate the risk of VTE based on the route and formulation of hormonal therapy. As shown, hormone therapy represents an array of medications that are each accompanied by their own profile of risks depending on the hormone dose, route of administration, and age of the patient. We hope that the growing body of evidence will calm fears of VTE risk to provide safe treatment of menopausal symptoms.
